# Larvicidal and repellent effects of essential oils on the brown dog tick (*Rhipicephalus sanguineus* Sensu lato) with description of new larval repellent activity test method

**DOI:** 10.1007/s10493-023-00892-2

**Published:** 2024-02-13

**Authors:** Samed Koc, Zeynep Nur Gultekin, Sevval Kahraman, Aysegul Cengiz, Burak Polat, Cansu Caliskan, Ozge Tufan-Cetin, Huseyin Cetin

**Affiliations:** 1 Faculty of Science, Department of Biology, Akdeniz University, Antalya, Turkey; 2 Laboratory Animals Application and Research Centre, Akdeniz University, Antalya, Turkey; 3 Department of Environmental Protection Technology, Vocational School of Technical Sciences, Akdeniz University, Antalya, Turkey

**Keywords:** Essential oil, *Origanum*, Repellent activity test, Ticks

## Abstract

The aim of this research was to investigate the larvicidal and repellent effects of essential oils (EOs) obtained from two Lamiaceae plant species, *Origanum minutiflorum* O. Schwarz & P.H. Davis and *Dorystoechas hastata* Boiss. & Heldr. ex Bentham, both endemic to Turkey, on *Rhipicephalus sanguineus* s.l. Latreille (Acari: Ixodidae). The study also introduces a new test method that can be used to assess the repellent effects against ticks. Both plant EOs exhibited the highest larvicidal activity against brown dog tick larvae after 24 h and LC_50_ and LC_90_ values were determined as 0.101% and 0.125% for *O. minutiflorum* essential oil and 0.937% and 2.1% for *D. hastata* essential oil, respectively. In this study, we have described a detailed protocol for a novel larval repellent activity test (LRAT) for essential oils and extracts, using simple equipment. The advantages and limitations of LRAT, when compared to other tests commonly used to determine repellent effect against ticks, are also included in this study. The LRAT was developed with modifications of the larval immersion test (LIT) and proves to be a highly efficient and easily observable method. It can be used to test any active substance that may be toxic to humans and animals. According to the LRAT, at the end of 3 h, *O. minutiflorum* essential oil showed a high repellent effect, varying between 84.14% and 100% at 1% concentration. This result was not statistically different from the DEET, the positive control. When comparing the larvicidal and repellent activities, *O. minutiflorum* essential oil was found to be more effective than *D. hastata* essential oil.

## Introduction

Diseases transmitted by vector organisms such as mosquitoes, ticks, sand flies and fleas are called vector-borne diseases (VBDs) and according to the reports of the World Health Organization, more than 17% of all the infectious diseases worldwide are transmitted by vectors (WHO 2020). Ticks are a group of arthropods that are main carriers of VBDs after mosquitoes in many parts of the world. Crimean-Congo hemorrhagic fever, Lyme disease, Rocky Mountain spotted fever, babesiosis, anaplasmosis, tick-borne encephalitis and Tularemia are examples of important diseases for which ticks are vectors (Jongejan and Uilenberg 2004).

*Rhipicephalus sanguineus* s.l. Latreille (Acari: Ixodidae), is known as the brown dog tick that is the most widespread tick in the world (Dantas-Torres 2010). This tick species was determined as a common species in public parks and city centers in Turkey (Koc et al. 2015; Aydın et al. 2020). Even though domestic dogs are the main host of *Rh. sanguineus* s.l., domestic cats, sheep, cattle, hedgehogs, foxes, and hares are able to be selected as host (Levin et al. 2013). This tick also is a vector of some zoonotic disease agents such as *Rickettsia conorii*, *Ehrlichia canis* and *Coxiella burnetii* (Dantas-Torres 2008). Topical treatment of hosts with chemicals and the use of collars with slow release acaricides are used for control of brown dog ticks. However, resistance to chemical acaricides developed by *Rhipicephalus* ticks reported by researchers in recent years (Eiden et al. 2015; Koc et al. 2022). In addition, chemical acaricides may cause toxic effects on dogs. Therefore, some acaricides may not be approved for use on dogs and other domestic pets by authorities (Adekoya et al. 2020; Salman et al. 2022).

Essential oils (EOs) extracted from different parts of flowering plants by water/steam distillation can be a source of eco-friendly and effective insecticides/acaricides alternatives to synthetic chemicals (Isman 2020). Plant EOs have long been used traditionally to protect animals and humans from parasites. The EOs contain a wide range of secondary metabolites such as terpenes, alkaloids, flavonoids and saponins that have many biological activities (Cavanagh and Wilkinson 2002; Sakkas and Papadopoulou 2017). In recent years, researchers have been focused on the acaricidal and repellent properties of EOs (Daemon et al. 2012; Araújo et al. 2016; Benelli and Pavela 2018). Many EOs have been found to be less toxic to humans and pets (such as dogs and cats) compared with conventional acaricides and EOs based formulations have now been used in numerous countries (Cetin et al. 2004; Isman 2020).

Therefore, in this research acaricidal and repellent activities of two endemic Lamiaceae species (*Origanum minutiflorum* O. Schwarz & P.H. Davis and *Dorystoechas hastata* Boiss. & Heldr. ex Bentham; Syn: *Salvia dorystoechas* B.T.Drew.) collected from Antalya - Turkey, were tested on brown dog tick, *Rh. sanguineus* s.l. This study also demonstrates for the first time a new Larval Repellent Activity Test (LRAT) method for the evaluation of repellent products.

## Materials and methods

### Tested plants and isolation of essential oil

Aerial parts of plants (*Dorystoechas hastata* Boiss. & Heldr. ex Bentham and *Origanum minutiflorum* O. Schwarz & P.H. Davis) used in this research, were collected (2 kg each) in the flowering period from their natural habitats in Antalya, Turkey. Voucher species were deposited in the Vector Ecology and Control Laboratory. Plants samples were dried two weeks in shade not exceeding room temperature and ground in a grinder to 2–4 mm diameter mesh size. After the plants dry, they were subjected to hydro-distillation for 2 h using a Clevenger-type apparatus and the methods described by Cetin and Yanikoglu (2006). Dried plant material (500 gr) was mixed with 2 L of tap water and boiled in a glass Clevenger apparatus. Based on the mass of dried plant material, the EO yield was calculated and expressed as percent (v/w). The EOs yields of *D. hastata* and *O. minutiflorum* were 2.4% and 2.9%, respectively. The EOs were stored in glass tubes at + 4 °C in a refrigerator until tested.

### Collection and identification of ticks

Adults of *R. sanguineus* s.l. ticks were collected without breaking their rostrum from the ears and head of domestic dogs in Antalya, Turkey. Ticks were identified by the first author of this article using the keys of Aydin (1994) and Aydin (2000). Adult female ticks were kept at 26–28 °C temperature, 80–90% relative humidity and 12:12 h light: dark photoperiod conditions for laying eggs. After fully engorged female ticks lay their eggs, eggs were placed in glass test tubes for hatching at the same temperature and humidity conditions as adults.

### Larval immersion tests (LIT)

The larvae emerged about 10–14 days after the eggs were laid. Larvae 12 to 15 days old were used in the experiments. Larvae that showed upward climbing and host-seeking behavior in the test tubes were used in the experiments. The essential oils were dissolved in Tween 80 (CAS No. 9005-65-6) solution prepared with distilled water. To determine the lethal concentration values, various concentrations (0.075-3% v/v) that cause 10–90% mortality in ticks were used in LIT tests. A package was formed by folding the filter paper (7.6 × 8.9 cm) (Whatman No. 1) and closing it with clips. Considering the size of the packages, 50–100 larvae were placed in each package using paint brushes. The packets were then immersed in tested concentrations for 5 min. After 5 min exposure, the packages were removed from the tested solutions and left to dry at room temperature. After 24 h the packages were opened, and the surviving/dead larvae were counted. Larvae, which does not react to the contact of the paintbrush under a stereomicroscope, were noted as dead. Because EOs can evaporate at room temperatures, LIT was performed instead of the larval packet test method. All experiments were carried out in triplicate and 0.3% Tween 80 solution was used as negative control, 0.2% Permethrin (CAS no. 52645-53-1) solution was used as positive control group. The larvicidal activity tests were conducted at 24 ± 2 °C temperature, 50 ± 10% relative humidity with a photoperiod of 12:12 h light and dark conditions.

### Larval repellent activity test (LRAT)

**Fig. 1:**
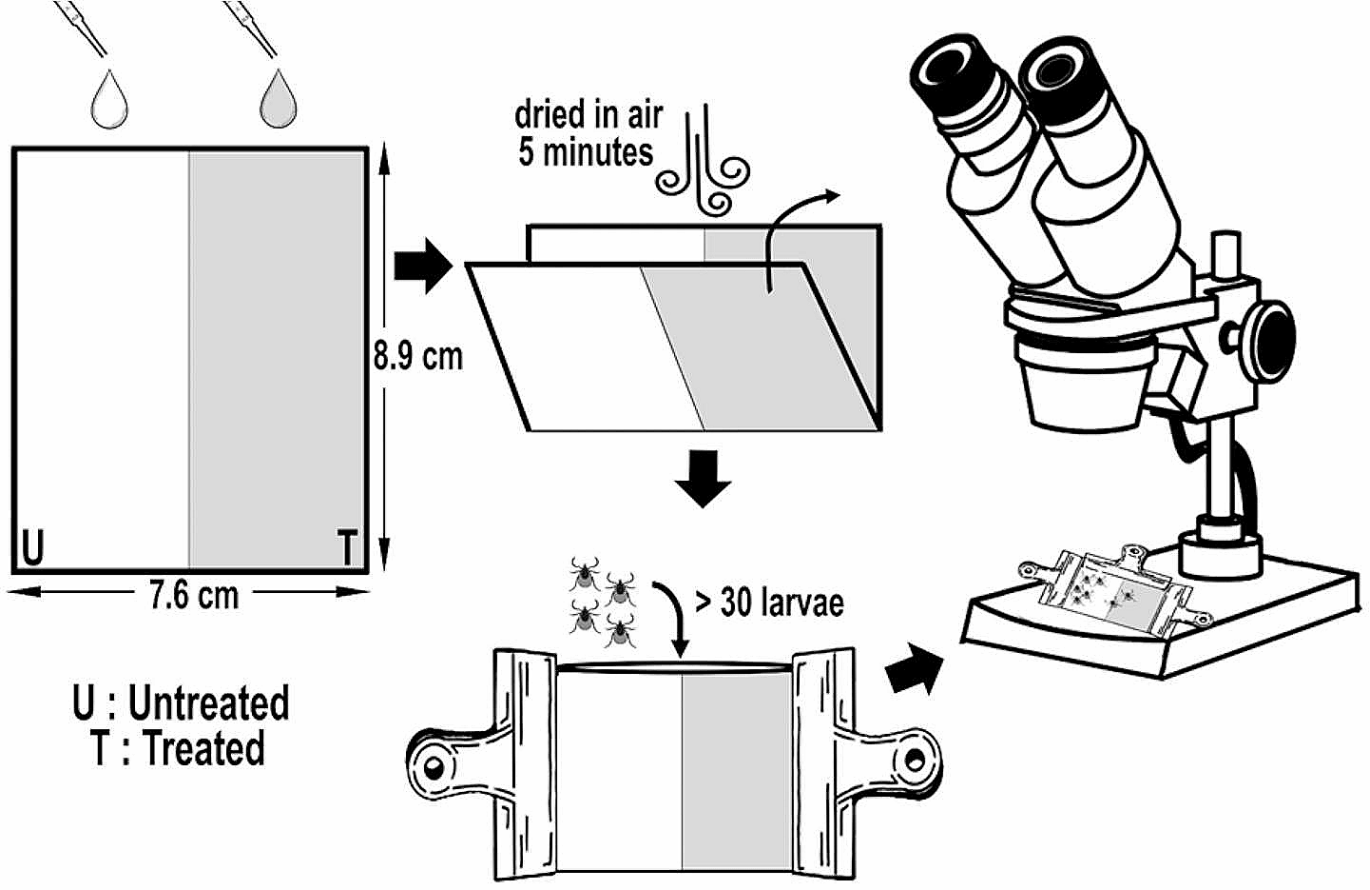
New larval repellent activity test (LRAT) method to evaluate repellent effects of active substances against ticks

**Fig. 2:**
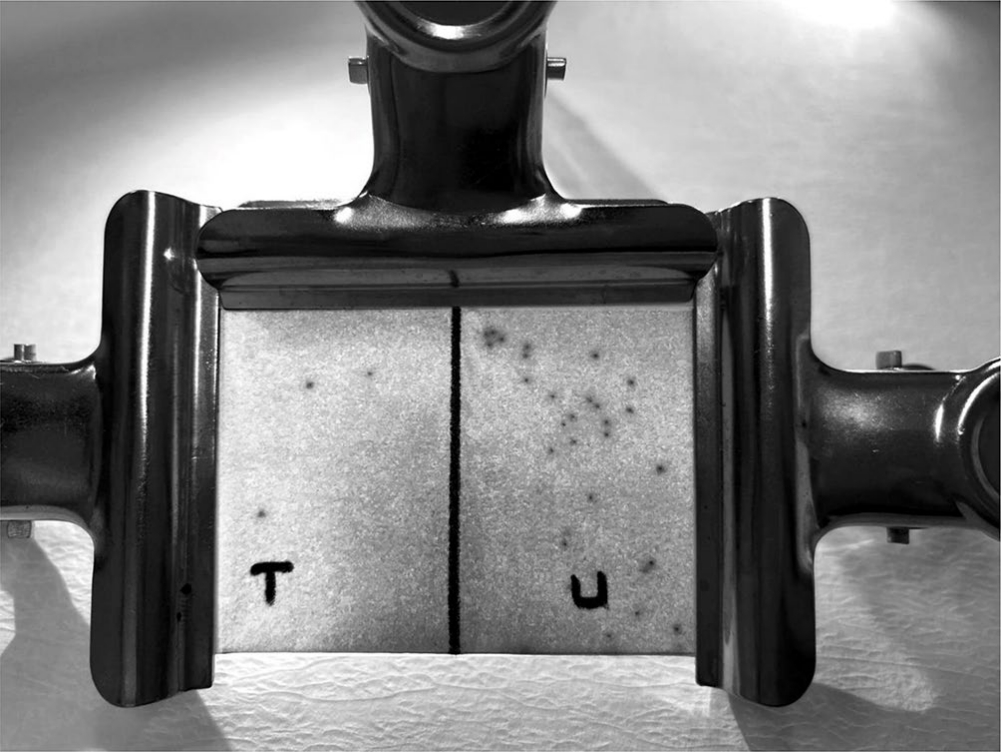
Appearance of ticks in the new larval repellent activity test (LRAT) method under the stereomicroscope

Three concentrations (0.1%, 0.5% and 1% v/v) of tested EOs were also used in repellency assays. A volume of 200 µl of test solution was applied to the half of the Whatman filter paper (No: 1) (7.6 × 8.9 cm) with even and complete coverage by pipettor and given 5 min to dry and it was folded. From folded filter papers was created a package by clips. Then using a paintbrush, more than 30 larvae (12–15 days old) were placed on each filter paper which has enough space for larvae (Fig. 1). Also, half of the packet has enough area for the larvae to move away from the repellent substances. Whatman filter papers treated with 0.3% Tween 80 solution alone was tested in untreated area as the negative control and treated with N, N-dietil m-toluamid (DEET15% CAS No. 134-62-3) in ethanol as positive control. The counts of the larvae in treated or untreated area in the packets were examined under the stereomicroscope and recorded at 1 h intervals until 6 h. In the repellent effect experiments, as the contact time increased, the tests were finalized after 6 h in the area where the essential oil was applied, since immobilization of the larvae was observed after 6 h. Ticks were considered repelled if they stayed on the untreated (control) area (Figs. 1 and 2). The following equation was used to calculate the percent repellency: Percent Repellency (%) = [(Ticks in control area-Ticks in treated area) / (Ticks in control area + Ticks in treated area)] × 100. All experiments were performed in triplicate. The repellent activity tests were conducted at 24 ± 2 °C temperature, 50 ± 10% relative humidity conditions.

### Statistical analysis

All statistical tests of our data were conducted using the SPSS 20.0 program. According to the Kolmogorov-Smirnov normality test all data shown normal distribution. Therefore, means were subjected to analysis of variance and data was compared with Duncan’s multiple range test (*p* ≤ 0.05). The larval mortality data were subject probit analysis and 50% lethal concentration (LC_50_) rates and 90% lethal concentration (LC_90_) rates with their confidence limits were determined.

## Results

The acaricidal (repellency and larval toxicity) effects of various concentrations of EOs of two endemic Lamiaceae species on *Rh. sanguineus* s.l., and the also acaricidal (repellency and larval toxicity) effects at various counting times are given in Tables 1 and 2. The obtained data clearly showed that *O. minutiflorum* essential oil was more effective than *D. hastata* essential oil in terms of repellent and larvicidal effects.

**Table 1:** Probit analysis of the larvicidal efficacy of tested plant essential oils against *Rhipicephalus sanguineus* s.l

Plant species	LC_50_ (%)	Confidence limits (%)	LC_90_ (%)	Confidence limits (%)	Chi-Square	df	P
*O. minutiflorum*	0.101	0.096–0.109	0.125	0.114–0.153	40.68	10	0.0001
*D. hastata*	0.937	0.764–1.176	2.1	1.584–3.384	83.97	10	0.0001

**Table 2:** Repellent effect of *Origanum minutiflorum* and *Dorystoechas hastata* essential oils at various concentrations against *Rhipicephalus sanguineus* s.l. tick (%repellency ± Standard Error)

Time (h)	Concentrations of *Origanum minutiflorum* essential oil			Control
	1%	0.5%	0.1%	DEET15%
1	100 ± 0.0 aB	91.74 ± 4.18 aB	38.41 ± 15.81 aA	95.73 ± 2.24 aB
2	84.14 ± 3.14 abB	83.42 ± 3.49 abB	47.25 ± 5.29 aA	100 ± 0.0 aB
3	89.7 ± 1.15 abB	84.4 ± 4.98 abB	54.59 ± 12.09 aA	100 ± 0.0 aB
4	74.24 ± 4.11 bA	72.82 ± 5.09 abA	40.04 ± 13.98 aA	100 ± 0.0 aB
5	77.41 ± 9.06 bA	69.04 ± 9.34 abA	73.02 ± 6.78 aA	100 ± 0.0 aB
6	77.52 ± 6.38 bB	64.87 ± 5.25 bAB	43.56 ± 3.99 aA	100 ± 0.0 aB
**Time (h)**	**Concentrations of ** ***Dorystoechas hastata*** **essential oil**			**Control**
	**1%**	**0.5%**	**0.1%**	**DEET15%**
1	41.67 ± 18 aA	60.42 ± 14.07 aA	53.33 ± 10.08 aA	95.73 ± 2.24 aB
2	48.79 ± 17.54 aA	51.23 ± 8.71 aA	48.61 ± 5.82 aA	100 ± 0.0 aB
3	43.01 ± 11.73 aA	58.61 ± 4.97 aA	30.26 ± 14.14 aA	100 ± 0.0 aB
4	47.3 ± 13.4 aA	72.22 ± 2.62 aA	59.93 ± 10.09 aA	100 ± 0.0 aB
5	65.79 ± 10.39 aA	72.24 ± 5.01 aA	44.26 ± 18.85 aA	100 ± 0.0 aB
6	59.45 ± 7.11 aA	72.07 ± 2.96 aA	55.93 ± 8.67 aA	100 ± 0.0 aB

There is no statistical difference if the lower case letters in the same column are the same for each time period (*p* > 0.05)

There is no statistical difference if capital letters are the same on the same line for each concentration (*p* > 0.05)

Both plant EOs exhibited the highest larvicidal activity against *R. sanguineus* s.l. larvae after 24 h (Table 1). When the LC_50_ and LC_90_ values obtained from two plant essential oils were compared, *O. minutiflorum* essential oil was found to be more effective than *D. hastata* essential oil. LC_50_ and LC_90_ values were determined as 0.101% and 0.125% for *O. minutiflorum* essential oil and 0.937% and 2.1% for *D. hastata* essential oil, respectively (Table 1).

In the larvicidal activity assays, positive control Permethrin caused 100% mortality and negative control Tween 80 caused 0% mortality. The larvicidal effect of *O. minutiflorum* is quite high. At a concentration of 0.1%, *D. hastata* caused no mortality, whereas the essential oil of *O. minutiflorum* caused more than 60% mortality. The essential oil of *O. minutiflorum* was as effective as permethrin at 0.25% concentration (cause 100% mortality), while *D. hastata* was only as effective at 3% concentration (cause > 94% mortality).

At the end of 3 h, *O. minutiflorum* essential oil showed high repellent effect varying between 84.14% and 100% at 1% concentration and was not statistically different (*p* > 0.05) from the DEET, positive control. At the end of 6 h, 1% concentration showed a minimum 74.24% repellent effect. *O. minutiflorum* essential oil showed a repellent effect varying between 41.67% and 65.79% at a concentration of 0.1% (Table 2). At a concentration of 0.5%, *O. minutiflorum* essential oil was statistically (*p* ≤ 0.05) effective as the positive control DEET in the first 3 h and showed a repellent effect ranging from 83.42 to 91.74%. At 0.5% concentration, there is no statistical difference in repellent effect between both oils at 4–6 h and it is less effective than the positive control (DEET) (Table 2).

*D. hastata* essential oil showed a repellent effect ranging from 30.26 to 72.24% at all concentrations for 6 h. DEET was statistically more effective than *D. hastata* oil at all times and concentrations (Table 2). Both EOs showed almost the same effect at all times at 0.1% concentration. When the different tested concentrations of the *O. minutiflorum* essential oil are compared among themselves, there is no significant difference between 0.5% and 1% concentrations in the first 3 h and the repellent effects of them are higher than 0.1% (the lowest) concentration. There was no statistically significant difference at all times between the three tested concentrations of *D. hastata* essential oil.

When the tested concentrations of both EOs were evaluated for repellency in terms of exposure times, there was no significant change in the repellent effect as time increased. Positive control DEET showed a repellent effect of 95.79–100% for 6 h. It was observed that the larvae showed normal walking behavior in both untreated and only Tween 80 applied packets, and they wandered freely in every part of the packets.

## Discussion

*Rhipicephalus* ticks, known as ectoparasites on domestic animals, are among the most common ticks in the world. Within this tick genus, *R*. *sanguineus* species known as dog tick has an important place. Although synthetic acaricides such as chlorpyrifos, fipronil, fluralaner, permethrin, sarolaner and selamectin etc. successfully have been used against ticks, according to many recent studies conducted in the world, the dog tick has developed resistance to various chemical acaricide groups in recent years (Eiden et al. 2015; Rodriguez-Vivas et al. 2017; Becker et al. 2019; Daniele et al. 2021).Plant-based pest control agents such as essential oils could provide an alternative because they have low environmental persistency. EOs obtained from flowering plants are important sources for the development of effective acaricides due to their low level of toxicity to humans and animals, high diversity of phenolic compounds and the synergistic relationships between the components (Salman et al. 2020; Selles et al. 2021). Many researchers have reported the toxic effects of EOs from plants in the Lamiaceae family on insects and ticks. The genus *Origanum* in the Lamiaceae plant family is one of the most researched genera. *Origanum bilgeri* essential oil at 0.8% concentration caused > 83% mortality on unfed *Rhipicephalus turanicus* Pomerantzev adults after 48 h (Koc et al. 2013). The toxic effects of *Origanum vulgare* EOs on *Haemaphysalis longicornis* tick were investigated by Qiao et al. (2021) and they found that *O. vulgare* oil showed high acaricidal activity in LITs. *R. turanicus* adults treated with 6.25% and higher concentrations of the essential oil from *Origanum onites* were dead 48 h following exposure (Coskun et al. 2008). In studies by which different *Origanum* species were tested on different ticks, mainly adult individuals were used. In our study, the tests were carried out on the larval stage and a very high effect (100%) was found at very low concentrations. Vapor phase toxicity of *O. minutiflorum* essential oil on *R. turanicus* adults was evaluated at a variety of concentrations (1–20 µl/L) and a variety of exposure times (30–120 min). The experiment with ticks exposed to vapors from cotton wicks containing at least 10 µl/L essential oil resulted in complete (100%) mortality at 120 min exposure time (Cetin et al. 2009).

Both tested endemic plant species in this research are used as food additives, spices and herbal teas in Turkey. Antimicrobial, antioxidant, insecticidal and acaricidal activities of EOs have been previously reported by researchers (Dadalioglu and Evrendilek 2004; Erkan et al. 2011; Oz et al. 2012; Selvi et al. 2022). Besides these studies, this is the first research to investigate the lethal and repellent effects of EOs obtained from the aerial parts of *D*. *hastata* and *O. minutiflorum* on brown dog tick *R. sanguineus* s.l. larvae. Furthermore, this paper describes a new method (LRAT) for testing products to be used as repellents against ticks. The LRAT method is safe and effective and it can be easily conducted without the need for human and animal subjects, using the same tools and equipment as the the LIT or LPT methods. It can be used to test any active substance that may be toxic to humans and animals. No ethics committee approval is required for these experiments. The LRAT method is convenient for observing ticks under stereomicroscope light and is applicable for assessing both synthetic repellents and essential oils, along with plant extracts. In the case of extracts that leave a color on Whatman filter paper in the treated area, it is easy to count the number of ticks in the untreated (uncolored) area at 1-h intervals. At the end of the test period (e.g. 4–8 h), place the test apparatus in the freezer for 30 min and count the total number of dead ticks. By subtracting the number of ticks in the untreated area from the total number of ticks, you can obtain the number of ticks in treated area for each time period. One important advantage of LRAT over other tests used to determine repellent effect is that it minimizes the possibility of ticks escaping from the test environment. The direction of the package formed for LRAT can be adjusted according to variable behavior of tick species. In methods such as the Petri dish method, it is difficult to observe the repellent effect, as the larvae are hidden on or under the application paper placed in the Petri dish. In addition, moving the petri dish while observing the repellency can causes ticks to escape and making it challenging to obtain accurate results. Compared to tube olfactometer tests, LRAT requires less space and allows for a large number of repetitions to be conducted simultaneously in laboratory conditions.

Many authors have previously reported major constituents of the EOs of both tested plants (Cetin et al. 2009; Oz et al. 2012; Albayrak and Aksoy 2019). Borneol, camphor and 1,8- Cineole were found to be the main constituents of *D. hastata* oil, while carvacrol was determined to be the main component of *O*. *minutiflorum* essential oil. Since carvacrol is present in large amounts in all *Origanum* essential oil contents, it has high levels of contact and fumigant toxicity on pests (Tabari et al. 2015; Xie et al. 2019). Especially the very high (> 80%) amount of carvacrol in the essential oil of *O. minutiflorum* and the fact that this component has very strong insecticidal and acaricidal activity may be the main reason for the high larvicidal and repellent effect of the essential oil. In this research, the toxic effects of tested essential oils on ticks are thought to result from contact toxicity, cytotoxicity and neurotoxicity (Selles et al. 2021).

## Conclusions

Our research findings showed that LRAT can be used for determination of repellent activity of various substances against ticks. *O*. *minutiflorum* and *D. hastata* essential oils, which exhibits toxicity (lethal and repellent) to the larvae of *Rhipicephalus sanguineus*, may be considered as potential acaricides.

## Data Availability

All data generated or analysed during this study are included in this published article.
